# Study of SME employees’ awareness level on lean manufacturing and ergonomics implementation in Malaysian and Indonesian production environments

**DOI:** 10.1016/j.heliyon.2024.e38216

**Published:** 2024-09-21

**Authors:** Ahmad Shah Hizam, Nik Mohamed, Nelfiyanti

**Affiliations:** a Faculty of Resilience, Rabdan Academy, 65, Al Inshirah, Al Sa'adah, Abu Dhabi, United Arab Emirates; b Faculty of Manufacturing and Mechatronic Engineering Technology, Universiti Malaysia Pahang Al-Sultan Abdullah, 26600, Pekan, Pahang, Malaysia; c Department of Industrial Engineering, Faculty of Engineering, Universitas Muhammadiyah Jakarta, 10510, Jakarta, Indonesia

**Keywords:** Ergonomics, Lean manufacturing, Linear regression, Small and medium enterprises (SMEs)

## Abstract

SMEs are small to medium-sized businesses with relatively fewer workers and lower revenues than large companies. However, SMEs also contribute to the local economic growth of a region, so the smooth production process needs to be considered to increase productivity. Applying lean manufacturing (LM) and Ergonomics concepts in the production process is critical because it can overcome smooth production and maintain the health and safety of SMEs workers. LM focuses on minimizing waste, while ergonomics focuses on humans as a source of energy in the smooth running of production activities. So, this study aims to measure the level of understanding of Malaysian and Indonesian SMEs workers on applying LM and Ergonomics concepts on the production floor and determine the effect of these two concepts using the SPSS and SmartPLS4 applications. SPSS serves to measure the validity, reliability and mean for the category of workers' understanding of LM and Ergonomics. SmartPLS4 helps us understand the influence of the two concepts. Based on the calculation of the mean for each variable of LM and ergonomics, it is found that Malaysian workers understand enough compared to Indonesian SMEs workers. As for the effect, Ergonomics has a significant influence on LM.

## Graphical abstract

**Figure:**
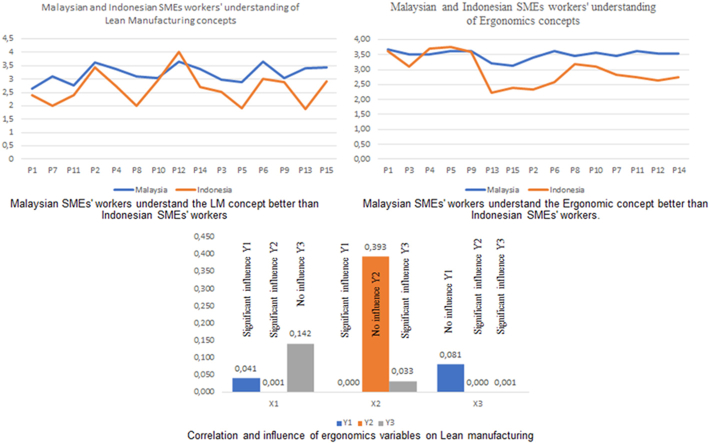

## 1 Introduction

Small and Medium Enterprises (SMEs) play an essential role in the economic growth of developing countries, including Malaysia and Indonesia. In both countries, SMEs contribute significantly to the Gross Domestic Product (GDP) and employment [[1], [2], [3], [4]]. Both countries have significant SMEs contributing to regional economic growth and job creation [5]. However, SMEs often face production efficiency and occupational safety challenges, affecting their competitiveness in the global market.

Lean manufacturing is a systematic approach to reducing waste in the production process and has been proven effective in improving productivity and operational efficiency [6]. LM is derived from Toyota's production system, which focuses on reducing waste and improving continuous value [7,8]. LM also aims to reduce production costs and enhance product quality and consumer satisfaction [9]. Meanwhile, ergonomics focuses on optimizing the interaction between humans and work systems, which contributes to improving worker safety, health, and performance [10]. Ergonomics is a science that studies humans as the primary system that interacts with their environment by considering and paying attention to the impact that will occur on humans [11,12]. Applying ergonomics is expected to improve workers' performance, safety and comfort at work [13,14]. Ergonomics focuses on designing workplaces, tools, and systems that meet the needs of physical and mental humans [12,15,16]. Integrating lean manufacturing and ergonomics has shown the potential to create a more productive and safer work environment [17].

Although the benefits of implementing lean manufacturing and ergonomics have been widely documented, the adoption rate among SMEs in Malaysia and Indonesia still needs to be higher [18,19]. One of the critical factors affecting the successful implementation of these concepts is the level of employee awareness and understanding [20].

Several previous studies have examined lean manufacturing implementation in Indonesian [21] and Malaysian [22] SMEs. However, these studies focus on technical and managerial aspects, focusing little on employee perception and awareness. Employees' understanding of lean principles and ergonomics is crucial for successful long-term implementation [23]. In the context of ergonomics, research in Indonesia has shown that awareness of ergonomics practices in SMEs is still limited, resulting in a high risk of occupational injuries and illnesses [24]. Despite improvements in the application of ergonomics principles in Malaysia, more understanding and implementation are still needed at the SME level [25].

Comparative studies between Malaysia and Indonesia in this regard are still rare, even though both countries have similar economic and cultural characteristics and differences in industrial policies and approaches to SMEs [26]. This comparison can provide valuable insights into the factors influencing employee awareness and effectiveness of lean manufacturing and ergonomics implementation in SMEs. Furthermore, recent developments in Industry 4.0 and digital transformation have brought new dimensions to applying lean manufacturing and ergonomics [27]. Employee awareness of integrating these technologies with lean and ergonomics principles is becoming increasingly crucial in SMEs striving to remain competitive in the digital age.

Therefore, this study aims to examine the level of awareness of SME employees in Malaysia and Indonesia regarding implementing lean manufacturing and ergonomics in the production environment. Workers' understanding was measured using the help of SPSS and SmartPLS4 applications. SPSS is a statistical tool used for descriptive statistical analysis, inferential analysis, multivariate analysis, data visualization and data management [[28], [29], [30]]. In this study, SPSS measures the validity and reliability of questionnaire items and the category of workers' understanding based on the mean value obtained. SMartPLS4 is software for partial least squares structural equation modelling, including path modelling, measurement model evaluation, structural model evaluation, mediation and moderation analysis, multi-group analysis, non-normal data, and visualization [31]. In this study, SmartPLS4 is used to determine the correlation and influence of ergonomics variables on lean manufacturing by the objectives of this study. This study will provide a deeper understanding of employee perceptions, implementation challenges, and potential strategies to increase the adoption of these practices in both countries.

## 2 Methodology

**Fig. 1:**
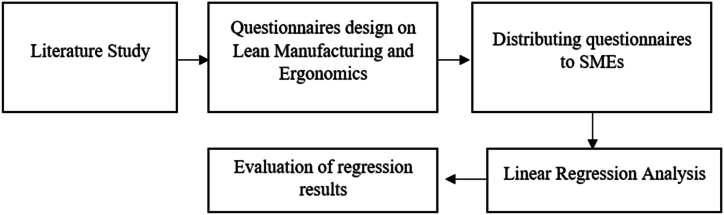
Flowchart of research methodology.

Fig. 1 depicts the flowchart of the methodology used in this study to achieve its objectives.

### 2.1 Literature review

Literature reviews were conducted to identify issues surrounding LM and ergonomics implementation. The methods comprise LM, ergonomics, and linear regression. LM and ergonomics were the variables used in achieving the study objectives. Linear regression was used in the data processing to analyze the achievement of the expected goals. LM was developed to maximize resource utilization by minimizing wastage [32]. LM is a powerful formula that can eliminate extravagance, control quality and inventory, improve overall performance, and provide high initial profits [33]. One of the factors found in LM is people, which is the most critical resource in a company. Humans perform physical tasks in assembling and handling materials, such as sensory and cognitive tasks in inspecting components, removing tools, entering data, and managing people and operations [34]. Ergonomics discusses the relationship between human workers, their tasks and jobs, and the design of things used [16]. Ergonomics is a science that studies human behavior regarding work and the adaptation of work tasks to human conditions to reduce future stress.

### 2.2 Questionnaires formulation

The questionnaires were formulated based on LM and ergonomics-related reviews. These questionnaires comprised 30 questions divided into the two methods used as variables. The questionnaires were divided into six variables (three LM and three ergonomic variables). The three LM variables comprised process flow management (Y1), efficient use of resources (Y2), and quality and process improvement (Y3). Meanwhile, the three ergonomic variables included work environment and employee performance (X1), stress and workload (X2), and personal protective equipment, work processes, and standards (X3). Each variable contained various questions spread randomly into 30 questions. Each question used a Likert scale category, which represents the worker's choice according to their feelings and understanding, comprising Very Low (1), Low (2), Medium (3), High (4), and Very High (5).

### 2.3 Distributing LM and ergonomics questionnaires

The formulated questionnaires were distributed to several SMEs in two countries, namely Malaysia and Indonesia, because most SMEs are found in these two countries. The distribution was made by randomly sampling several types of SMEs, such as food, metal, furniture, and others. The number of respondents who answered the LM and ergonomics questionnaire comprised 56 employees of Malaysian SMEs and 41 employees of Indonesian SMEs. The next step involved workers in each SME filling out the questionnaires based on the conditions faced in their daily activities. The completed questionnaires were distributed to owners and employees of SMEs to determine their level of understanding of LM and ergonomics applications. Each respondent answered the questions according to their knowledge, understanding, and the conditions they felt. Each question was formulated using plain language that was easy for SME employees to understand, making it easier for them to complete the questionnaire.

### 2.4 Linear regression analysis

Before the linear regression analysis was performed, validating each question found in each variable Y (ergonomics) and variable X (LM) using the SPSS application was necessary. Validation is to prove whether each material, process, procedure, activity, system, equipment, or mechanism used in production and supervision consistently yields the desired result. Verification and validation activities are essential to ensure the intended purpose and use [35], as the variables employed markedly influence workers' satisfaction and complaints during the work process. The validity test assesses the correlation between each indicator item score and the total construct score, using a significance level of 0.05 (5 %), yielding the following results [29]: if the value of Sig. (2-tailed) <0.05 and Pearson's correlation is positive, then the question item is declared valid and vice versa if the value of Sig. (2-tailed) > 0.05 is declared invalid. Additionally, Ho (null hypothesis) can be accepted when the observed correlation coefficient (r count) exceeds the critical value (r table), indicating that the measuring instrument used is valid or authentic [36,37].

After the validity test comes a reliability test that complements the testing process. The reliability test measures the questionnaire's consistency, which indicates the variable or construct of this research. Questionnaires are considered reliable if individuals provide consistent answers to the questions over time [30,38]. The reliability coefficient α exceeding 0.7 suggests sufficient reliability. If α > 0.80, then all items are considered reliable, and the entire test consistently demonstrates strong reliability. This interpretation is commonly understood as follows: α > 0.90, 0.70 ≤ α ≤ 0.90, 0.50 ≤ α ≤ 0.70, and α < 0.50 imply that reliability is perfect, high, moderate, and low, respectively. Low α suggests that one or more query items are not reliable [39].

The results of the validity and reliability testing of the questionnaire items, which are crucial for achieving the research objectives, showed that the obtained data are valid and consistent. Consequently, they can be effectively utilized in the subsequent stage, which involves assessing the comprehension of Malaysian and Indonesian SME employees regarding LM and ergonomics implementation in their workplace. Testing was done with the statistical calculation of the mean section using SPSS software. The mean measures data centrality. The mean is a representative of a group of data or an average value considered closest to the actual measurement results [40,41].

Regression analysis, which assesses the relationship between the dependent (y) and independent (x) variables, determines how changes in the independent variable affect the dependent variable. The amount of contribution of variable x to variable y is indicated by the relationship expressed in a mathematical equation, which illustrates the functional relationship between the two variables. The general equation of linear regression is y = a + bx [42].

Where:

Y = regression line/variable response

a = constant

b = regression constant

x = independent variable.

This regression analysis was carried out using the SMartPLS4 application. SmartPLS4 is a software for variant-based structural equation modelling (PLS-SEM) [43]. SmartPLS models relationships between variables and measures constructs in social and business research [31,43].

### 2.5 Evaluation of regression results

The final step of the processing stage entails evaluating linear regression results to achieve the study objectives: the level of workers’ understanding of both variables. At this stage, solutions are devised to address the awareness levels of SME workers regarding LM and ergonomics. Hence, a proposal is formulated to introduce an ergonomic standard operating procedure for work processes aimed at mitigating safety and health complaints.

## 3 Results and discussion

### 3.1 Validity and reliability testing process

SPSS testing was employed to assess the validity of each LM and ergonomics question, analyzing the Pearson correlation value and comparing the observed R count with the critical R table value. R count exceeding the R table implies that the questionnaire is valid. The table is obtained by determining the value of degree of freedom (DF) with the formula (df = n−2), where n is the sample size at a significant level of 5 %. The R table value used in this study is 0.2632 (df = 56 − 2 = 54, with 5 % significance). The calculated R-value for each item of the LM and ergonomics questions falls in 0.453–0.7287, which exceeds the table's R-value of 0.2632. This finding indicates that 15 questions about LM and 15 questions about ergonomics are valid. These questions were then used for the next stage in achieving the study purpose.

After the validity test comes reliability testing, which measures the consistency of the questionnaire variables. Table 1, Table 2 contain the reliability testing results for the LM and ergonomics questionnaires.

**Table 1:** Results of reliability testing of LM questionnaires using SPSS.

Cronbach's	Cronbach's Alpha Based on Standardized Items	N of Items
0.747	0.883	16

**Table 2:** Results of reliability testing of the ergonomics questionnaires using SPSS.

Cronbach's	Cronbach's Alpha Based on Standardized Items	N of Items
0.750	0.900	16

The obtained Cronbach's alpha for the LM questionnaire is 0.883, indicating that all question items are highly reliable and consistent.

The observed Cronbach's alpha for the ergonomics questionnaires is 0.900, which has a significant value of 0.80, indicating that all items are reliable and consistently contribute to robust evaluation. The validity and reliability results demonstrated that all LM and ergonomics question items were “valid” and consistent” and thus could be used for the following process: testing the level of understanding of SME employees toward LM and ergonomics.

### 3.2 Assessing SME employees’ understanding level of LM and ergonomics application

Based on the calculation of the mean statistical item for each question found in each variable, the analysis results of Malaysian and Indonesian SME employees’ understanding of LM and ergonomics application are listed in Table 3, Table 4.

a. Process flow management (Y1)

**Table 3:** LM concept understanding assessment.

Country	Likert Scale Y1 (PFM)				Likert Scale Y2 (EUR)							Likert Scale Y3 (QPI)						
	Questionnaires			Mean	Questionnaires						Mean	Questionnaires						Mean
	P1	P7	P11		P2	P4	P8	P10	P12	P14		P3	P5	P6	P9	P13	P15	
Malaysia (N = 56)	2.64	3.09	2.75	2.83	3.63	3.38	3.09	3.05	3.66	3.36	3.36	2.98	2.89	3.66	3.04	3.41	3.43	3.24
Indonesia (N = 41)	2.39	2.00	2.34	2.24	3.44	2.73	2.00	2.93	4.00	2.71	2.97	2.51	1.90	3.00	2.88	1.88	2.90	2.51

**Table 4:** Ergonomics concept understanding assessment.

Country	Likert Scale X1(WEP)								Likert Scale X2(SW)					Likert Scale X3(PWS)				
	Questionnaires							Mean	Questionnaires				Mean	Questionnaires				Mean
	P1	P3	P4	P5	P9	P13	P15		P2	P6	P8	P10		P7	P11	P12	P14	
Malaysia (N = 56)	3.68	3.50	3.50	3.61	3.61	3.21	3.11	3.46	3.39	3.61	3.46	3.55	3.50	3.45	3.61	3.52	3.52	3.52
Indonesia (N = 41)	3.61	3.10	3.71	3.76	3.59	2.22	2.39	3.20	2.32	2.59	3.17	3.10	2.79	2.83	2.73	2.63	2.73	2.73

Three questions included understanding product production, transferring materials and semifinished products/finished products, and unprocessed materials. The mean value obtained based on the three questions for Malaysian SME employees was 2.83, which fell in the “low understanding” category. In comparison, Indonesian SME employees had a mean value of 2.24, which also fell in the “low income' category. Overall, these findings suggest that employees of Malaysian and Indonesian SMEs both need a better understanding of the LM concept of process flow management.

b. Efficient use of resources (Y2)

The LM section for the efficient use of resources (Y2) comprises six questions about production planning, storage capacity, distance of movement influence on material handling, movement of workers, availability of facilities at the place of activity, and excessive activity. Based on the six questions, the mean value for Malaysian SME employees was 3.36, which fell in the “moderate understanding” category. A mean value of 3.20 was obtained based on the values given by Indonesian SME employees, which also fell in the “low understanding” category. This finding indicates that, for the LM Y2 variable, Malaysian workers understand better than Indonesian workers.

c. Quality and process improvement (Y3)

Six questions for variable Y3 contained information about the pile of materials, quality of materials, application of quality control, addition of movement in activities, the need for sorting, and time standards in each activity. Based on the question, a mean value of 3.36 was obtained, which fell in the “moderate understanding” category for all Malaysian SME employees. In contrast, Indonesian SME employees were included in the “low understanding” category with a mean value of 2.97. Malaysian SME employees better understand the LM concept for variable Y3 than do Indonesian SME employees.

d. Work environment and employee performance (X1)

Variable X1 is part of ergonomics, comprising seven questions about workspace temperature, work environment, needs, owner support, cooperation, ergonomic work procedures, and ergonomic workload standards. Based on the seven questions, a mean value of 3.46 was obtained, included in the “moderate understanding” category for all Malaysian SME employees. The Indonesian SME employees had a mean value of 3.20, which was also included in the “moderate understanding” category. Malaysian and Indonesian SME employees both have a moderate understanding of the concept of ergonomic variable X1.

e. Stress and workload (X2)

Variable X2 has four questions about work stress, workload of each employee, impact of the workload and workload on the production target. A mean value of 3.50 was obtained, which fell in the “moderate understanding” category for Malaysian SME employees. In comparison, Indonesian SME employees had a mean value of 2.79, which fell in the “low understanding” category. This finding suggests that Malaysian SME employees better understand the ergonomic concept of variable X2 than Indonesian SME employees.

f. Work processes and standards (X3)

The ergonomic concept for the X3 variable comprises four questions that contain understanding: employee posture in each activity, procedures for using personal protective equipment, ergonomic work procedures, and practical working hour standards. All Malaysian and Indonesian SME employees assessed inquiries based on their understanding, yielding evaluation results. Malaysian SME employees exhibited a mean understanding of 3.52, classified as “moderate,” whereas Indonesian counterparts displayed a mean understanding of 2.73, categorized as “low.” This finding suggests that Malaysian SME employees have a better understanding of the ergonomic concept of variable X3 than do Indonesian SME employees.

The analysis of mean values using the SmartPLS4 application, based on evaluations from Malaysian and Indonesian SME employees, revealed that Malaysian SMEs have a superior understanding of LM and ergonomics compared to their Indonesian counterparts, who, on average, are categorized as not understanding the concepts.

### 3.3 Linear regression analysis

Linear regression analysis shows the closeness of the causal relationship between variables and can be used to make predictions. This study applied linear regression to determine the direction and extent of the influence of the independent variable on the dependent variable using SmartPLS4 software. A linear regression assessment can use the standard reference values and information in Table 5 to determine the relationship between variables.

**Table 5:** Value standards and information from linear regression results [44].

Criteria	Value	Description
R-Square	0.25	Weak Model
	0.50	Medium model
	0.75	Strong Model
F-Square	0.02	Small
	0.15	Secondary
	0.35	Big
Path Coefficients	P Value < 0.05	Significant Influence
	P Value > 0.05	Not Significantly Affected

The multiple linear regression process in this research comprises the following: work environment and employee performance (X1), stress and workload (X2), personal protective equipment and work procedures (X3), process flow management (Y1), efficient use of resources (Y2), and quality and process improvement (Y3). Based on these variables, this study's multiple linear regression calculation process can be made using the final value reference in Table 5 to determine the relationship. The process of calculating the multiple linear regression of variable X on variable Y can be described below.

a. The relationship between X1, X2, and X3 against the Y1 Model

**Fig. 2:**
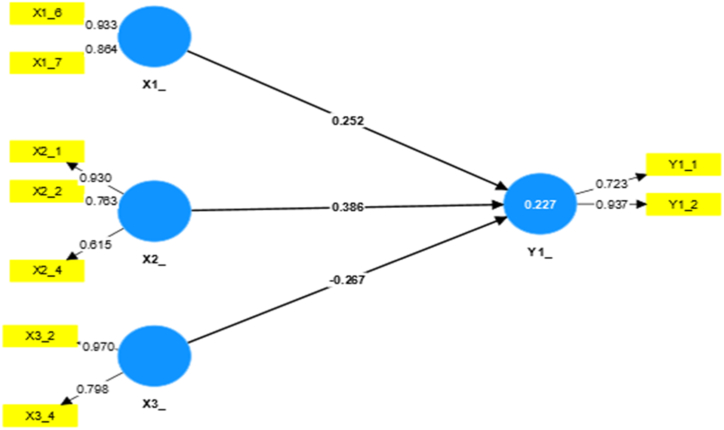
Relationship between variables X1, X2, and X3 with Y1.

Fig. 2 shows the multiple linear regression calculations using SmartPLS to determine the relationship between variables X1, X2, and X3 with Y1. Several questions are missing because they have a value < 0.60, which means invalidity, including X1-1, X1-2, X1-3, X1-4, X1-5, X2-3, X3-1, and X3-3. The rest have a value > 0.60, which means validity, and can be used to determine the relationship.

As seen in Fig. 2, the R-square-overview value of the relationship between the three X variables to Y1 is 0.227, which means “weak.” As for the relationship of each variable X to Y1, the total effect values are 0.252 (X1_Y1), 0.386 (X2_Y1), and −0.267 (X3_Y1). This means that X1 and X2 to Y1 have a positive value with a “weak” relationship category, and X3 to Y1 is included in the “no relationship” category because it has a negative value. Table 6 contains the results of the effect of each variable X on Y1 based on the calculation results.

**Table 6:** Results of the calculation of the P value of the effect of each variable X on Y1.

	P value	Standardized P value (influential)	Category of influence
X1_Y1	0.041	<0.05	Significant influence
X2_Y1	0.000		Significant influence
X3_Y1	0.081		No influence

Based on Fig. 2 and Table 6, variables X1 and X2 significantly influence Y1, with a “weak” relationship. Variable X3 does not influence Y1 with the category “no relationship.”

b. Relationship between X1, X2, and X3 with Y2 Model

**Fig. 3:**
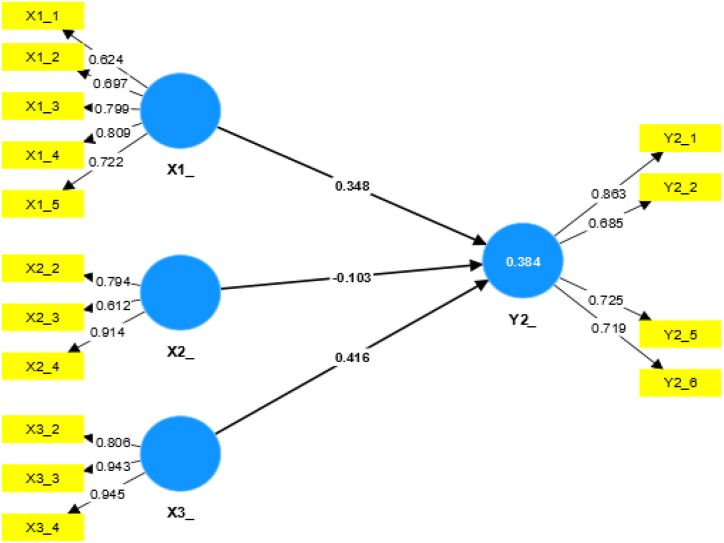
Relationship between variables X1, X2, and X3 with Y2.

Fig. 3 shows the relationship between variables X1, X2, and X3 with variable Y2. The outer loading value is valid if it exceeds 0.60. If the external loading value is below 0.60, the data are deleted (the deleted data X1: questions 6 and 7, X2: questions 1, X3: question 1 and Y2: questions 3 and 4). The R-square value obtained for the relationship of the three X variables with Y2 is 0.384, which is included in the “weak” relationship category. Table 7 shows the relationship between the influence of each variable X on Y2.

**Table 7:** Results of the calculation of the P value of the effect of each variable X on Y2.

	P value	Standardized P value (influential)	Category of influence
X1_Y2	0.001	<0.05	Significant influence
X2_Y2	0.393		No influence
X3_Y2	0.000		Significant influence

As listed in Table 7, X1 and X3 have a “significant effect” on Y2 because they have a P value < 0.05. Meanwhile, X2 “has no influence” on Y2 because it has a value > 0.05. Based on Fig. 3 and Table 7, variable X has a “weak” relationship to Y2 with a “significant influence.”

c. The relationship between X1, X2, and X3 with Y3 Model

**Fig. 4:**
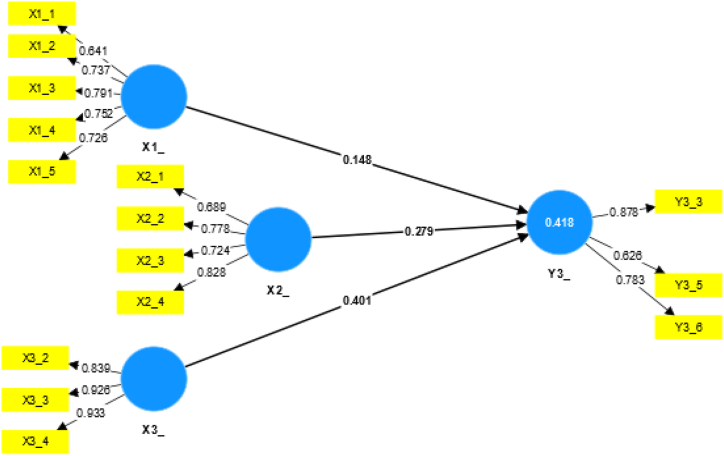
Relationship between variables X1, X2, and X3 with Y3.

Fig. 4 illustrates the relationship between variables X1, X2, and X3 with variable Y3, along with the validity calculation value for each variable based on individual questions. A variable is declared valid if its outer loading value > 0.60; if < 0.60, the variable/data should be deleted. Based on the results of the calculation, (1) delete questions 6 and 7 in Variable X1, (2) delete question 1 in Variable X3, and (3) delete questions 1, 4 and 2 in Variable Y3. Therefore, the result of calculating the relationship between the three variables X1, X2, and X3 against Y3 has a weak relationship model. This weak relationship is obtained from the calculation of the R-Square value of 0.418 with a positive value for the relationship of the three X variables to Y3 for the total effects value comprising 0.148 for X1 to Y3, 0.279 for X2 to Y3, and 0.401 for X3 to Y3. The relationship between the influence of each variable X on Y3 is listed in Table 8.

**Table 8:** Results of the calculation of the P value of the effect of each variable X on Y3.

	P value	Standardized P value (influential)	Category of influence
X1_Y3	0.142	<0.05	No influence
X2_Y3	0.033		Significant influence
X3_Y3	0.001		Significant influence

As listed in Table 8 for the effect of each variable X on Y3, X2 and X3 have a significant effect on Y3, while X1 has no significant effect on Y3. This finding shows that variable X has a “weak” relationship and a “significant effect” on variable Y3.

### 3.4 Evaluation of regression results

Based on the results of the multiple linear regression calculation of the relationship between the three variables X to each of Y1, Y2, and Y3, the three variables (X) have a weak relationship to each Y type. This differs from the level of influence of each variable X to each of Y (Y1, Y2, and Y3); that is, (1) the influence of X1 and X3 on Y1 falls within the weak influence category, while X2 and Y1 have a moderate influence, (2) the influence of X1 and X2 on Y2 falls within the weak influence category, while that of X3 on Y2 has a moderate influence, and (3) the influence of X1 and X2 on Y3 falls within a weak influence, while X3 has a moderate influence on Y3. This means that variable X has a weak relationship with variable Y and has an influence that is divided into small and medium categories. In this case, modifying questions with interrelated aspects is crucial to simplifying the understanding of the correlation and influence between LM variables and ergonomics.

### 3.5 Social impact of measuring SMEs workers' understanding of LM and ergonomics

Measuring SME workers' understanding of lean manufacturing and ergonomics concepts has significant social impacts. First, increased worker awareness of work efficiency and safety can drive positive behavioural changes, improving productivity and well-being [45]. It aligns with the findings of [17], which showed that implementing lean manufacturing in SMEs can increase job satisfaction and reduce workers' stress levels.

Furthermore, a better understanding of ergonomics can reduce the risk of occupational injuries and improve workers' quality of life. A study by Ref. [46] revealed that integrating lean principles and ergonomics can create a safer and more productive work environment. However, this implementation process can also present social challenges [47]. found that resistance to change and knowledge gaps between generations of workers can hinder the adoption of lean practices in SMEs.

From a managerial perspective, measuring worker understanding can help identify skill gaps and training needs [48]. Emphasized the importance of customized training programs to improve the effectiveness of lean implementation in SMEs. Furthermore [49], showed that improving workers' understanding of lean and ergonomics can reinforce a positive work culture and increase employee engagement.

Social impacts are also seen in a broader context. SMEs that adopt lean practices and ergonomics tend to be viewed as more professional by society and stakeholders [50]. It can increase the competitiveness of SMEs and create more employment opportunities in local communities. However [51], warns that improper implementation can lead to social tensions and workplace dissatisfaction.

Finally, knowledge transfer from the workplace to the community is also a significant social impact. Workers who understand lean concepts and ergonomics can become agents of change in their communities, spreading more efficient and healthy work practices [52]. This can raise public awareness about occupational safety and health more broadly.

## 4 Conclusion

Measurement of the understanding of Malaysian and Indonesian SMEs workers on the application of LM and Ergonomics concepts using the help of SPSS and SmartPLS4 applications obtained results: (a) All LM and Ergonomics question items are declared valid and reliable with an r-count > t-table value (>0.2632). It can be used for the measurement process. (b) Based on the measurement results using the help of SMartPLS4, it is found that Malaysian SMEs workers understand the concepts of LM and Ergonomics better than Indonesian SMEs workers. It can be interpreted that Indonesian workers need to learn or understand more about these two concepts than Malaysian SME workers, who already know and understand them quite well. (c) The correlation between ergonomics and LM is weak, but ergonomics significantly affects LM in its application on the production floor, with a P-value below 0.05.

## CRediT authorship contribution statement

**Ahmad Shah Hizam:** Investigation, Funding acquisition. **Nik Mohamed:** Writing – review & editing, Methodology, Conceptualization. **Nelfiyanti:** Writing – original draft, Validation, Software, Methodology, Formal analysis, Writing – original draft, Validation, Software, Methodology, Formal analysis.

## Declaration of competing interest

The authors declare that they have no known competing financial interests or personal relationships that could have appeared to influence the work reported in this paper.
