# Supplementary material for: Study of SME employees’ awareness level on lean manufacturing and ergonomics implementation in Malaysian and Indonesian production environments
Source: Heliyon. 2024 Sep 21;10(18):e38216. doi: 10.1016/j.heliyon.2024.e38216 (PMC11447361; doi:10.1016/j.heliyon.2024.e38216)
Supplement: Multimedia component 1 [file mmc1.docx]

Attachment 1.

Lean Manufacturing Questionnaire (against the seven wastes)

**Lean Manufacturing Application Questionnaire in Small and Medium Industries (SMIs)**

Sincerely,

Assalammualaikum Wr Wb

Let me introduce Nelfiyanti, one of the research team from the Faculty of Manufacturing and Mechatronic Engineering Technology Universiti Malaysia Pahang. I want to conduct a research on: “**Situation Analysis and Current Conditions of Lean Manufacturing and Ergonomics Implementation in Small and Medium Industries (Case Study: Several Indonesian and Malaysian SMIs)**”. For that, we need data for related information about the application of those two things by distributing the Lean Manufacturing and Ergonomics Application questionnaire to the owners and employees of **SMIs**, who will be the respondents in this research.

For this reason, we hope you will be willing to fill out this questionnaire as data that we will use in this research. We hope all the information provided follows the current situation and conditions at the SMI you belong to and where you work.

Thank you very much.

Researcher,

(Nelfiyanti)

**Respondent data**

Name :

Nature of the company

(i.e Food, logistic, packaging, etc :

Age :

Working hours :

Put a mark (x) on each question you choose based on the situation and conditions in the Small and Medium Enterprises (SMEs) where you work.

Information and Description:

**Lean manufacturing**

Lean Manufacturing is a production system that emphasises waste elimination by involving all the workers in the company. The intended waste is all actions that do not provide added value. Lean Manufacturing consists of seven wastes that the companies should eliminate. The seven wastes consist of:

1. Waste of Overproduction

It happens because there is an advantage in production, both in the form of finished goods and semi-finished goods with access order.

1. Waste of Inventory

This occurs because inventory is one of the effects of excessive production waste. Finished goods, semi-finished goods and excess materials at all stages of production will require storage, significant capital and additional energy to supervise them.

1. Waste of Defects

It happens because the quality of the product is not good or bad, so it needs repair and causes the accumulation of additional costs.

1. Waste of Transportation (Transfer/Transportation)

Occurs due to poor or bad production layout, poor organization in the workplace, which has an impact on the activity of moving goods from one place to another.

1. Waste of Motion

It occurs due to the unnecessary movement of workers or machines that do not have added value to the product. An example is placing a component far from the employee's reach so that it requires more movement for the recruitment process.

1. Waste of Waiting

This occurs because workers, both human and machine, are idle. They are waiting due to machine damage, late supply of components, loss of work tools or waiting for results and information.

1. Waste of Over processing

Occurs due to excessive activity that does not provide added value. Examples are the checking process that is done repeatedly, the approval process that goes through several stages and others.

**Questions about Lean Manufacturing**

1. Do SMIs produce semi-finished products/finished goods in large quantities without orders from consumers?

| 1. Never | 1. Sometimes | 1. Enough | 1. Often | 1. Definitely |
| --- | --- | --- | --- | --- |

1. Does SMIs have production planning in producing the type of product accurately?

| 1. Never | 1. Sometimes | 1. Enough | 1. Often | 1. Definitely |
| --- | --- | --- | --- | --- |

1. Are there unnecessary material piles around the material storage area?

| 1. Never | 1. Sometimes | 1. Enough | 1. Often | 1. Definitely |
| --- | --- | --- | --- | --- |

1. Is the size of the storage area sufficient so that overload capacity does not occur and to avoid congestion from the warehouse line?

| 1. Never | 1. Sometimes | 1. Enough | 1. Often | 1. Definitely |
| --- | --- | --- | --- | --- |

1. Are materials, finished goods and semi-finished goods damaged during processing or transfer for further processing?

| 1. Never | 1. Sometimes | 1. Enough | 1. Often | 1. Definitely |
| --- | --- | --- | --- | --- |

1. Has Quality Control been implemented at every stations of the current processes (even for the unnecessary checking)?

| 1. Never | 1. Sometimes | 1. Enough | 1. Often | 1. Definitely |
| --- | --- | --- | --- | --- |

1. Is there a transfer of materials, semi-finished goods and finished goods from the warehouse to the production section/product delivery section that is done many times outside the existing standards?

| 1. Never | 1. Sometimes | 1. Enough | 1. Often | 1. Definitely |
| --- | --- | --- | --- | --- |

1. Is the material storage warehouse, semi-finished goods and finished goods have a long distance so that it requires a longer and longer transfer/transportation process?

| 1. Never | 1. Sometimes | 1. Enough | 1. Often | 1. Definitely |
| --- | --- | --- | --- | --- |

1. During the work process, do the workers require extensive movements in taking materials and tools to be used?

| 1. Never | 1. Sometimes | 1. Enough | 1. Often | 1. Definitely |
| --- | --- | --- | --- | --- |

1. Does the employee perform the Movement of stepping from the current work position in taking materials and semi-finished goods to be processed?

| 1. Never | 1. Sometimes | 1. Enough | 1. Often | 1. Definitely |
| --- | --- | --- | --- | --- |

1. Are there many materials, finished goods and semi-finished goods waiting to be processed and sent to consumers, even though they do not have order?

| 1. Never | 1. Sometimes | 1. Enough | 1. Often | 1. Definitely |
| --- | --- | --- | --- | --- |

1. Is the equipment available and ready to be used for the next available process?

| 1. Never | 1. Sometimes | 1. Enough | 1. Often | 1. Definitely |
| --- | --- | --- | --- | --- |

1. Is the material often mixed with semi-finished goods or finished goods so that it requires sorting for the next process?

| 1. Never | 1. Sometimes | 1. Enough | 1. Often | 1. Definitely |
| --- | --- | --- | --- | --- |

1. Are the current work procedures capable of eliminating unnecessary and redundant work?

| 1. Never | 1. Sometimes | 1. Enough | 1. Often | 1. Definitely |
| --- | --- | --- | --- | --- |

1. Does SMIs have a standard time used for each activity or job?

| 1. Never | 1. Sometimes | 1. Enough | 1. Often | 1. Definitely |
| --- | --- | --- | --- | --- |

Description:

If Answer: “**Never” then value 1**; **“Sometimes” then has a value of 2**; **“Enough” worth 3;**

**“Often” then value 4, and “Definitely” then value 5.**

**Ergonomics.**

Ergonomics is a science that discusses design for humans which is interpreted as an effort made in adapting the work environment to the needs of users or humans. Ergonomics has the purpose of increasing productivity and reducing discomfort while working.

Below are questions about activities related to employees in their work environment. As for the questions, they consist of:

1. Does a work space with cool air make me more skilled in doing my job?

| 1. Very low | 1. Low | 1. Medium | 1. High | 1. Very high |
| --- | --- | --- | --- | --- |

1. Do employees during the work process experience stress due to the mismatch between the work process and the actual one that occurs?

| 1. Very low | 1. Low | 1. Medium | 1. High | 1. Very high |
| --- | --- | --- | --- | --- |

1. Does the current work environment make the employee comfortable in doing work or make the employee experience discomfort that affects the employee's performance?

| 1. Very low | 1. Low | 1. Medium | 1. High | 1. Very high |
| --- | --- | --- | --- | --- |

1. Are all the needs required by the employees well met?

| 1. Very low | 1. Low | 1. Medium | 1. High | 1. Very high |
| --- | --- | --- | --- | --- |

1. Does the support from SMIs owners have a good impact on employee performance?

| 1. Very low | 1. Low | 1. Medium | 1. High | 1. Very high |
| --- | --- | --- | --- | --- |

1. Do employees have an unbalanced workload due to an inappropriate number of employees?

| 1. Very low | 1. Low | 1. Medium | 1. High | 1. Very high |
| --- | --- | --- | --- | --- |

1. Does the worker carry out the activity of lifting, taking and placing materials/finished goods/semi-finished goods from the floor to the next activity with the best possible procedure for the worker without considering the further impact on the worker's body parts?

| 1. Very low | 1. Low | 1. Medium | 1. High | 1. Very high |
| --- | --- | --- | --- | --- |

1. How is the workload felt by the employees in meeting production targets?

| 1. Very low | 1. Low | 1. Medium | 1. High | 1. Very high |
| --- | --- | --- | --- | --- |

1. Do the employees at SMIs help each other and provide a sense of comfort for each employee so as to have an impact on the smoothness of the production process?

| 1. Very low | 1. Low | 1. Medium | 1. High | 1. Very high |
| --- | --- | --- | --- | --- |

1. How is the workload felt by employees when instructed or assigned by the leader?

| 1. Very low | 1. Low | 1. Medium | 1. High | 1. Very high |
| --- | --- | --- | --- | --- |

1. Does SMIs have a procedure for using personal protective equipment to avoid work accidents?

| 1. Very low | 1. Low | 1. Medium | 1. High | 1. Very high |
| --- | --- | --- | --- | --- |

1. Does SMIs have work procedures that have considered ergonomic factors so that they can overcome the pain complaints felt by employees?

| 1. Very low | 1. Low | 1. Medium | 1. High | 1. Very high |
| --- | --- | --- | --- | --- |

1. Do SMIs have information on how to work ergonomically that is displayed in the work area?

| 1. Very low | 1. Low | 1. Medium | 1. High | 1. Very high |
| --- | --- | --- | --- | --- |

1. Do SMIs have effective standard hours in the production process?

| 1. Very low | 1. Low | 1. Medium | 1. High | 1. Very high |
| --- | --- | --- | --- | --- |

1. Do SMIs have a minimum ergonomic workload standard that will be burdened for employees?

| 1. Very low | 1. Low | 1. Medium | 1. High | 1. Very high |
| --- | --- | --- | --- | --- |

Description:

If Answer: “**Never low” then value 1**; **“Low” then has a value of 2**; **“Medium” worth 3;**

**“High” then value 4, and “Very high” then value 5.**
